# Interference with Glycosaminoglycan-Chemokine Interactions with a Probe to Alter Leukocyte Recruitment and Inflammation *In Vivo*

**DOI:** 10.1371/journal.pone.0104107

**Published:** 2014-08-05

**Authors:** Sandra Li, Ulrika S. Pettersson, Bart Hoorelbeke, Elzbieta Kolaczkowska, Katrien Schelfhout, Erik Martens, Paul Kubes, Jo Van Damme, Mia Phillipson, Ghislain Opdenakker

**Affiliations:** 1 Laboratory of Immunobiology, Rega Institute for Medical Research, KU Leuven, Leuven, Belgium; 2 Department of Medical Cell Biology, Uppsala University, Uppsala, Sweden; 3 Laboratory of Virology and Chemotherapy, Rega Institute for Medical Research, KU Leuven, Leuven, Belgium; 4 Jagiellonian University, Krakow, Poland; 5 Snyder Institute for Chronic Diseases, and Department of Physiology and Pharmacology, University of Calgary, Calgary, Canada; 6 Laboratory of Molecular Immunology, Rega Institute for Medical Research, KU Leuven, Leuven, Belgium; Università degli Studi di Milano, Italy

## Abstract

*In vivo* leukocyte recruitment is not fully understood and may result from interactions of chemokines with glycosaminoglycans/GAGs. We previously showed that chlorite-oxidized oxyamylose/COAM binds the neutrophil chemokine GCP-2/CXCL6. Here, mouse chemokine binding by COAM was studied systematically and binding affinities of chemokines to COAM *versus* GAGs were compared. COAM and heparan sulphate bound the mouse CXC chemokines KC/CXCL1, MIP-2/CXCL2, IP-10/CXCL10 and I-TAC/CXCL11 and the CC chemokine RANTES/CCL5 with affinities in the nanomolar range, whereas no binding interactions were observed for mouse MCP-1/CCL2, MIP-1α/CCL3 and MIP-1β/CCL4. The affinities of COAM-interacting chemokines were similar to or higher than those observed for heparan sulphate. Although COAM did not display chemotactic activity by itself, its co-administration with mouse GCP-2/CXCL6 and MIP-2/CXCL2 or its binding of endogenous chemokines resulted in fast and cooperative peritoneal neutrophil recruitment and in extravasation into the cremaster muscle *in* *vivo*. These local GAG mimetic features by COAM within tissues superseded systemic effects and were sufficient and applicable to reduce LPS-induced liver-specific neutrophil recruitment and activation. COAM mimics glycosaminoglycans and is a nontoxic probe for the study of leukocyte recruitment and inflammation *in* *vivo*.

## Introduction

Chemokines are established central players in coordinating directional and selective leukocyte migration into tissues for immune regulation in physiology and pathologies, including inflammatory disorders, infection and cancer. These chemotactic cytokines have emerged to constitute a large family of over 50 different members, all of which are characterized by their small sizes (∼8 to 10 kDa) and related structures [1], [2]. Accordingly, chemokines are segregated into C, CC, CXC and CX_3_C subfamilies, based on the arrangement of conserved NH_2_-terminal cysteine motifs [3]. For most chemokines a further biological distinction can be made between homeostatic or constitutively expressed chemokines, and inflammatory or inducible chemokines. The latter subclass is expressed by non-immune and immune cells upon induction by various stimuli, including cytokines, such as IL-1β, TNF-α and IFN-γ, and microbial-derived molecular patterns [4]. Among inflammatory chemokines the ELR^+^ CXC chemokines carry an NH_2_-terminal conserved ELR (Glu-Leu-Arg) motif preceding the first cysteine amino acid residue, important in receptor interactions. These ELR^+^ CXC chemokines are predominantly responsible for neutrophil chemotaxis, which is initiated through binding of the G protein-coupled seven-transmembrane domain receptors CXCR1 and/or CXCR2, and also possess angiogenic features [5], [6]. Examples of ELR^+^ CXC chemokines are human IL-8/CXCL8, described as the most powerful chemoattractant for human neutrophils, and murine “granulocyte chemotactic protein-2” (GCP-2)/CXCL6, “keratinocyte-derived chemokine” (KC)/CXCL1 “and macrophage inflammatory protein-2” (MIP-2)/CXCL2 as potent neutrophil chemokines in the mouse [7]. On the contrary, CXC chemokines that lack the ELR motif, such as “monokine induced by IFN-γ” (MIG)/CXCL9, “IFN-γ-inducible protein of 10 kDa” (IP-10)/CXCL10 and “IFN-inducible T cell α chemoattractant” (I-TAC)/CXCL11, are predominantly chemotactic toward activated T lymphocytes, NK cells and monocytes, and additionally contain angiostatic properties [6]. CC chemokines, including “monocyte chemotactic protein-1” (MCP-1)/CCL2, “macrophage inflammatory protein-1α” (MIP-1α)/CCL3 and “regulated upon activation, normal T cell expressed and secreted” (RANTES)/CCL5 are predominantly chemotactic for monocytes, NK cells, dendritic cells and activated T cells [1], [2]. Furthermore, chemokines tend to synergize directly or indirectly with other chemokines, cytokines, inflammatory mediators or pathogen-derived molecules, thereby providing a powerful mechanism to strengthen leukocyte recruitment [8]–[10].

Besides mediating their biological effects by binding to chemokine receptors, another interaction of chemokines implicates binding to linear sulphated glycosaminoglycan chains (GAGs), including heparan-, dermatan- and chondroitin sulphates and heparin. Leukocyte recruitment into tissues is supposed to require chemokine presentation on endothelial cells to circulating immune cells, as well as the establishment of a sustained chemotactic gradient across the vessel walls and into the extracellular matrix and tissues. GAGs are crucial molecules throughout this process by locally restraining chemokines, preventing chemokine dilution and even protection against proteolysis [11], [12]. Indeed, heparan sulphate has been shown to immobilize chemokines at the luminal endothelial cell surface [13]. In particular, sequestering of MIP-2/CXCL2 on endothelial cells through heparan sulphate binding is essential to establish intraluminal crawling and endothelial transmigration of neutrophils [14]. However, additional tools are needed to study chemotaxis *in* *vivo* and to evaluate whether and how interference with chemotaxis may be possible, beneficial or detrimental.

Recently, we have described a polyanionic polysaccharide derivative, designated COAM (for chlorite-oxidized oxyamylose), as an immunomodulator with antiviral activity [15], [16]. When injected intraperitoneally (i.p.), COAM induces the recruitment of neutrophils and macrophages that are in part essential to control viral burden and mortality upon acute infection with a neurotropic virus [16]. This effect of COAM, mediated by binding of GCP-2/CXCL6, illustrates that neutrophils contribute to antiviral resistance by the host, and can be further exploited to combat acute neuroinflammation [17], and cancer [18].

The negative charges and linear structure, together with the binding characteristics for GCP-2/CXCL6, provide COAM with features resembling natural GAGs. In fact, COAM possesses higher affinities to mouse GCP-2/CXCL6 than heparan sulphate and chondroitin sulphate [16]. This fact incites systematic investigations about local and general effects of COAM on leukocytes. For instance, it is not known whether and which other chemokines than GCP-2/CXCL6 interact with COAM, whether this interaction is different from that with heparan sulphate and what these findings clarify about the biological effects of COAM *in* *vivo*.

Here, we compared the binding of various mouse chemokines to COAM and heparan sulphate, a prototypic GAG. We showed that COAM had the ability to bind specifically to chemokines with kinetics similar to or higher than those for heparan sulphate. This information suggested that COAM might compete with GAGs for selective chemokine binding. The chemokine binding feature of COAM was translated to *in* *vivo* local recruitment of leukocytes, as COAM synergized with GCP-2/CXCL6, MIP-2/CXCL2 and endogenous chemokines for recruitment of neutrophils in different animal models of local application. In addition, this formed the basis to use COAM-induced chemotaxis and to test its effects on systemic neutrophil recruitment. We thus established that COAM is a critical probe to study cell recruitment *in* *vivo*.

## Materials and Methods

### Reagents

COAM was synthesized by a two-step oxidation of amylose, purified and fractionated according to molecular weight (MW) as described previously [15], [19]. COAM was endotoxin-free and either used as a MW mixture or as high MW fractions (corresponding to protein molecular equivalent weights exceeding 100 kDa). Poly(I:C) and heparan sulphate were purchased from Sigma-Aldrich (St. Louis, MO). Because many heparan sulphate preparations are heterogeneous and may vary from batch to batch, we used a heparan sulphate preparation with similar molecular characteristics as COAM, as detailed previously [18]. In addition, previously we also compared COAM with other glycosaminoglycans, including chondroitin sulphate and heparan sulphate preparations for binding to GCP-2/CXCL6 and demonstrated higher affinities for COAM than for both glycosaminoglycans [16]. Recombinant mouse chemokines were obtained from Peprotech (Londen, UK).

### Mice

Animal experiments at the Rega Institute for Medical Research, University of Leuven, were carried out with female adult NMRI mice purchased from Elevage Janvier (Le Genest Saint Isle, France), in agreement with the Ethical Committee for Animal Care and Use of the KU Leuven (License number for Belgium LA1210243) and with adherence to international guidelines for animal ethics and welfare. Mouse studies at the University of Uppsala were performed in adult C57BL/6 male mice purchased from Taconic (M&B, Ry, Denmark) and housed in the local animal facility under standardized conditions of temperature (21–22°C) and illumination (12 h light/12 h darkness) with free access to tap water and pelleted food (Type R36, Lantmännen, Kimstad, Sweden). The experiments were approved by the Regional Animal Ethics Committee in Uppsala, Sweden. For the intravital microscopy studies, male C57Bl/6J mice were purchased from Jackson Laboratories (Bar Harbor, ME). Animals were maintained in a specific pathogen-free environment at the University of Calgary Animal Resource Centre. All experimental animal protocols were approved by the University of Calgary Animal Care Committee and were in compliance with the Canadian Council for Animal Care Guidelines.

### Surface plasmon resonance (SPR)

Binding kinetics of chemokines to COAM and heparan sulphate were determined by surface plasmon resonance (SPR) analysis on a Biacore T200 instrument (GE Healthcare, Uppsala, Sweden). COAM was fractionated by gel filtration chromatography on Superdex S-200 (GE Healthcare) [15] and high MW COAM was biotinylated, as follows. COAM and heparan sulphate were dissolved at 2 mg/ml in 0.15 M sodium chloride, 0.1 M sodium phosphate pH 7.5. Biotinamidohexanoic acid hydrazide (Sigma-Aldrich) at a final concentration of 5 mM was reacted with COAM for 2 h at room temperature. Excess reactant was removed by gel filtration on a 10 ml Sephadex G-25 column equilibrated with PBS [20]. Biotinylated high MW COAM and heparan sulphate were immobilized on a streptavidin (SA)-coated biosensor chip (GE Healthcare). A reference flow cell was used as a control for non-specific binding and refractive index changes. All interaction studies were performed at 25°C. The tested chemokines were serially diluted in HBS-P (10 mM HEPES, 150 mM NaCl and 0.05% surfactant P20; pH 7.4) using two-fold dilution steps. Samples were injected for 2 min at a flow rate of 45 µl/min and dissociation was followed for 5 min. Several buffer blanks were used as double referencing controls. Regeneration of the SA sensor chip surface was performed with a 1 second pulse of 50 mM NaOH. Experimental data were fit by using the 1∶1 binding model with mass transfer correction (Biacore T200 Evaluation software 1.0) to determine the binding kinetics. Affinities (K_D_) were estimated from the ratio of dissociation (k_off_) and association (k_on_) rate constants.

### Leukocyte recruitment into the peritoneal cavity

NMRI mice were i.p. injected with 1 mg COAM (high MW; 200 µl) or 100 ng mouse GCP-2_(9–78)_ (200 µl), or with a solution of premixed COAM and mouse GCP-2_(9–78)_, all diluted in sterile endotoxin-free PBS. At various time points after injection, peritoneal cells were collected by washing the peritoneal cavity with 5 ml PBS containing 2% FBS. Total cell numbers from these lavage fluids were determined and single cell suspensions containing 0.5×10^6^ cells were stained with FITC-conjugated anti-Ly6G mAb (clone 1A8) and APC-conjugated anti-CD11b mAb (eBioscience, San Diego, CA) for the presence of neutrophils. Samples were analyzed with a FACSCalibur flow cytometer (BD Biosciences Immunocytometry Systems, San Jose, CA) by using CellQuest software. Absolute cell numbers were calculated by multiplying the obtained cell percentages with total peritoneal cell counts.

### *In vivo* neutrophil recruitment into the cremaster muscle

Male C57Bl/6 mice of similar age were injected with an intrascrotal injection of 200 µl with either saline (sterile NaCl) or COAM (0.2 mg) 3 or 24 h prior to experiments. At the time for experiment, mice were anaesthetized by spontaneous inhalation of isoflurane gas (Forene, Abbott Scandinavia AB, Stockholm, Sweden) via an isoflurane pump (Univentor 400 Anesthesia Unit, AgnTho’s AB, Lidingö, Sweden) through a breathing mask containing a mixture of air and oxygen (total oxygen 40%) and ∼2.4% isoflurane. The animals were placed on a water-heated operating table to maintain body temperature at ∼37°C. The depth of anesthesia was controlled by regularly monitoring peripheral reflexes. The cremaster muscle was prepared as previously described [21], [22]. Briefly, the muscle was dissected free from other tissues and opened longitudinally with cautery. The muscle was held flat on a cover slip by attaching five sutures in the corner of the tissue and the tissue was then constantly superperfused (1 ml/min) with pre-warmed bicarbonate buffered saline (pH 7.4), throughout the experiment. After a resting period of 30 min, a cremasteric venule with a diameter of ∼25–35 µm was selected and its blood flow was recorded during a 5-minute-period through an intravital microscope (Leica Microsystems DM5000B, Wetzlar, Germany) with a CCD camera (Hamamatsu Orca-R2, Hamamatsu City, Japan) connected to a computer with Volocity 5.0 Acquisition software. After the first recording period, a low dose (0.5 nmol/l) of the chemokine macrophage inflammatory protein-2 (MIP-2/CXCL2; R&D Systems, Abingdon, UK) was added to the superperfusate in some of the groups throughout the remaining experiment. MIP-2 binds to the receptor CXCR2 and has previously been shown to recruit predominantly neutrophils [21], and the low dose of this chemokine was chosen to clearly reveal a possible COAM-potentiating effect on numbers of recruited neutrophils. Periods of five minutes were recorded at 30, 60 and 90 min after MIP-2 addition. The number of rolling leukocytes was counted during each of these 5 min periods and an average of rolling neutrophils per min was calculated. The rolling cell velocity of the first ten rolling cells during each period was measured. Also, the number of adherent neutrophils in a 100 µm long segment of the venule, as well as the number of emigrated cells in the field of view (FOV) (200 µm×300 µm, 0.06 mm^2^) was analyzed. The cremaster muscles were saved for further analyses.

### Preparation of the mouse liver for intravital microscopy

Mice received an i.p. injection of 1 mg/kg LPS 4 hr prior to intravital microscopy. Alternatively, some animals received an injection of LPS (1 mg/kg) and COAM (2 mg/mouse). Mice were anesthetized with a mixture of ketamine hydrochloride (200 mg/kg, Rogar/SBT) and xylazine hydrochloride (10 mg/kg, MTC Pharmaceuticals). After anesthesia, cannulation of the right jugular vein was performed for administration of additional anesthetic and for injection of antibodies or other reagents. Preparation for intravital imaging of the liver was performed as previously described [23]. Briefly, a midline incision followed by a lateral incision along the costal margin to the midaxillary line was performed to expose the liver. The mouse was placed in a right lateral position, and ligaments attaching the liver to the diaphragm and the stomach were cut, thus allowing the liver to be externalized onto a glass coverslip located on the inverted microscope heat-controlled stage. Exposed abdominal tissues were covered with saline-soaked gauze to prevent dehydration. The liver was draped with a saline-soaked tissue paper to avoid tissue dehydration and to help restrict movement of the tissue on the slide.

### Spinning disk confocal intravital microscopy

The exposed liver lobe was visualized with an Olympus IX81 inverted microscope equipped with a confocal light path (Wave-Fx; Quorum) based on a modified Yokogawa CSU-X1 head (Yokogawa Electric Corporation) with a UPLANSAPO 10×/0.40 or UPLANSAPO 20×/0.70 air objective. Four laser excitation wavelengths (491, 561, 643, and 730 nm; Cobalt) were used in rapid succession and visualized with the appropriate long-pass filters (Semrock). Exposure times for excitation wavelengths were 400 ms for all lasers. A back-thinned EMCCD 512×512 pixel camera (C9100–13, Hamamatsu, Bridgewater, NJ) was used for fluorescence detection. Volocity acquisition software (Improvision) was used to drive the microscope.

### Analysis of spinning disk confocal microscope-acquired images

Fluorescence imaging of neutrophil counts and NET components was performed with intravital immunofluorescence analysis. Neutrophils were visualized by injection of Alexa-fluor 750-anti-mouse Ly6G antibody (3 µg). Extracellular DNA was labeled with Sytox Green DNA dye (5 µM), histone H2Ax was labeled with Alexa-fluor 555-anti-mouse H2Ax antibody (5 µg), and neutrophil elastase (NE) was labeled with Alexa-fluor 647-anti-mouse NE antibody (0.6 µg). All antibodies and dyes were injected i.v. 15 min prior to intravital imaging. Neutrophils and NETs were quantified with SD-IVM using previously published methodology [23]. In brief, images were acquired as z stacks of xy planes (1 µm intervals) from the bottom to top of sinusoids in each field of view using a 20× objective lens, and saved as extended focus images in.tiff format. Images from individual color channels (e.g., red for histone H2Ax, far red for elastase) were exported and analyzed in ImageJ (NIH). Neutrophils were counted per 10×FOV, minimum 4 FOV from each mouse. Intensity of histone and elastase staining was analyzed so that differences in background fluorescence between experiments and antibody lots could be accounted for and background autofluorescence could be eliminated. Contrast was adjusted to minimize autofluorescent background staining, and a minimum brightness threshold was set to yield only positive staining. The same contrast and threshold values were applied to all images from all treatment groups within the experiment. Thresholded images were converted to binary (black and white), and the area per field of view covered by positive fluorescence staining (black) was calculated with ImageJ software. Data were expressed as the percentage of area in each FOV covered by positive fluorescence staining.

### Statistical analysis

Statistical analyses were performed using Prism 5 (GraphPad Software, San Diego, CA) or SigmaStat 3.5 (Systat Software, Richmond, VA). Differences between treatment and control groups were evaluated using the nonparametric Mann-Whitney *U* test for comparing two groups, or the Kruskal-Wallis test for comparing three or more groups. For the *in* *vivo* neutrophil recruitment experiments, one way repeated measurements of ANOVA was used when comparing the same animal at different time points with Dunnett’s post hoc test and student’s t-test was used for comparing two groups. *P* values<0.05 were considered statistically significant.

## Results

### Mouse CXC and CC chemokines bind to COAM and heparan sulphate

**Figure 1:**
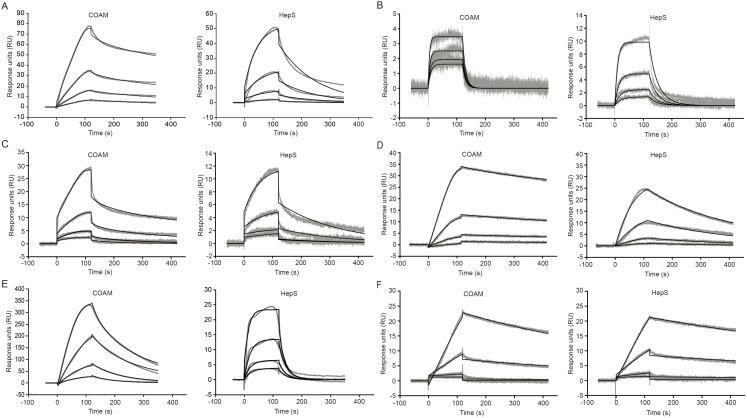
Binding of mouse chemokines to COAM and heparan sulphate. Concentration ranges from 200*o*f mouse (**A**) GCP-2_9–78_, (**B**) KC/CXCL1, (**C**) MIP-2/CXCL2, (**D**) IP-10/CXCL10, (**E**) I-TAC/CXCL11 and (**F**) RANTES/CCL5 was run over SA sensor chips surfaces onto which biotinylated COAM and heparan sulphate (HepS) were immobilized. Binding was measured by SPR technology and the resulting experimental sensorgrams are shown in gray. For curve fitting, shown as black lines, the following concentrations were used in two-fold dilution series: 50–6.25 nM for GCP-2_9–78_, 25–3.13 nM for KC/CXCL1, 200–25 nM for MIP-2/CXCL2, 6.25–0.78 nM for IP-10/CXCL10, 50–6.25 nM for I-TAC/CXCL11 and 400–50 nM for RANTES/CCL5.

Several chemokines have already been shown to bind with varying affinities to glycosaminoglycans (GAGs), including heparan sulphate. These interactions and their selectivities co-determine chemokine function and regulation [11]. Previously, we showed that COAM binds to mouse GCP-2/CXCL6 with higher affinity than heparan sulphate and chondroitin sulphate and that the resulting *in* *vivo* recruitment of neutrophils partially explains host antiviral resistance. Because neutrophil depletion did not completely wipe out the antiviral effect [16], we hypothesized that other leukocyte types and, hence, other chemokines might be involved. We here investigated systematically whether COAM displays GAG-mimetic functions, by measuring interactions of various mouse chemokines with COAM and comparing these with heparan sulphate, using SPR technology. We used COAM- and also heparan sulphate-mediated binding of the mouse neutrophil chemoattractant GCP-2/CXCL6, as illustrated in Fig. 1A as a reference experiment [16]. A truncated form of mouse GCP-2/CXCL6, GCP-2_(9–78)_, was used as the reference, as this processed form results in a marked potentiation of neutrophil chemotaxis compared with the intact form, both *in* *vitro* and *in* *vivo* [24]. Decreasing concentrations (two-fold dilutions, starting from 200 nM) of soluble recombinant GCP-2_(9–78)_ resulted in a concentration-dependent binding interaction between GCP-2_(9–78)_ and immobilized COAM and heparan sulphate. Next, the binding of other chemokines to COAM was specified. Two other mouse ELR^+^ CXC chemokines, KC/CXCL1 and MIP-2/CXCL2, and the mouse ELR^−^ CXC chemokines IP-10/CXCL10 and I-TAC/CXCL11 efficiently bound to both COAM and heparan sulphate in a concentration-dependent manner (Fig. 1B–E). Due to non-specific binding interaction with the reference flow channel, binding of the mouse ELR^−^ CXC chemokine MIG/CXCL9 resulted in abnormal binding curves for COAM and heparan sulphate (data not shown). Of the four mouse CC chemokines (MCP-1/CCL2, MIP-1α/CCL3, MIP-1β/CCL4, RANTES/CCL5) that were tested, only RANTES/CCL5 displayed binding interactions with COAM and heparan sulphate (Fig. 1F), whereas MCP-1/CCL2, MIP-1α/CCL3 and MIP-1β/CCL4 were found not to bind to either COAM or heparan sulphate even at the highest concentration tested (400 nM) (data not shown). Since the CC chemokines MCP-1/CCL2, MIP-1α/CCL3 and MIP-1β/CCL4 did not bind, whereas RANTES/CCL5 did, we deduced that selectivity existed in chemokine binding to both COAM and heparan sulphate. As previously shown for the binding of GCP-2/CXCL6 to heparan sulphate and chondroitin sulphate, the binding intensity for specific chemokines differed considerably between COAM and heparin sulphate (*vide infra*).

### Kinetic analysis of the interaction between mouse chemokines and COAM or heparan sulphate

Examination of the obtained sensorgrams suggested different binding characteristics between different mouse chemokines for the same compound and also between the two compounds for the same chemokine. Association and dissociation phases were measured and the resulting sensorgrams were used for calculating kinetic parameters to characterize the nature of the binding interactions (Table 1). For KC/CXCL1, both the association (k_on_) and dissociation (k_off_) rates were comparable between COAM and heparan sulphate, which resulted in corresponding mean affinity K_D_ values of 13.1±1.0 nM and 12.1±0.7 nM, respectively. MIP-2/CXCL2 displayed a fast association and dissociation rate towards COAM, whereas binding to heparan sulphate was characterized by a 2.3 log slower association and a 1.7 log slower dissociation. This resulted in a>3-fold stronger affinity of MIP-2/CXCL2 toward COAM (K_D_ = 20.7±4.9 nM) compared to heparan sulphate (K_D_ = 78.0±15.6 nM). Moreover, the sensorgrams for MIP-2/CXCL2 showed a biphasic response, i.e. an initial rapid association and dissociation rate were followed by a reduction of these rates. GCP-2/CXCL6 displayed a tight affinity toward COAM (K_D_ = 1.1±0.6 nM) and a 2-log difference in association rates resulted in a 86.6 fold weaker affinity toward heparan sulphate (K_D_ = 95.3±6.1 nM). Also I-TAC/CXCL11 showed a 7.7-fold tighter affinity for COAM (K_D_ = 15.3±1.1 nM) in comparison to heparan sulphate (K_D_ = 118.3±53.3 nM). Of all chemokines tested, IP-10/CXCL10 showed the strongest affinity for COAM and heparan sulphate with comparable mean K_D_ values of 0.41±0.08 nM and 0.95±0.08 nM, respectively. The 10-fold difference between the k_off_ values resulted in a faster dissociation rate for heparan sulphate compared to COAM. The mouse CC chemokine RANTES/CCL5 showed the weakest binding affinity for COAM and heparan sulphate, with a 3-fold stronger binding to heparan sulphate compared to COAM. Together, these results indicated that COAM mimics glycosaminoglycans by binding to chemokines, the interaction of which is characterized by high affinity in the nanomolar K_D_ range. We were able to order the K_D_ values for COAM as follows, from high to low affinity: IP-10/CXCL10> GCP-2/CXCL6> KC/CXCL1> I-TAC/CXCL11> MIP-2/CXCL2> RANTES/CCL5. For heparan sulphate affinity, these chemokines were ranged in the following order: IP-10/CXCL10> KC/CXCL1> MIP-2/CXCL2> GCP-2/CXCL6> I-TAC/CXCL11> RANTES/CCL5. This comparison indicated that the interactions between chemokines and COAM *versus* heparan sulphate were different.

**Table 1:** Kinetic parameters resulting from SPR analysis with COAM and heparan sulphate versus different mouse chemokines.

	COAM			Heparan sulphate		
Chemokine	k_on_ (1/M.s)	k_off_ (1/s)	K_D_ (nM)	k_on_ (1/M.s)	k_off_ (1/s)	K_D_ (nM)
**KC/CXCL1**	(1.49±0.92) E+06	(1.90±1.06) E−02	13.1±1.0	(1.57±0.02) E+06	(1.91±0.13) E−02	12.1±0.7
**MIP-2/CXCL2**	(1.21±1.02) E+07	(2.24±1.53) E−01	20.7±4.9	(5.96±1.91) E+04	(4.52±1.12) E−03	78.0±15.6
**GCP-2/CXCL6**	(8.73±4.77) E+06	(8.24±0.23) E−03	1.1±0.6	(9.09±2.00) E+04	(8.61±1.37) E−03	95.3±6.1
**MIG/CXCL9**	N.D.	N.D.	N.D.	N.D.	N.D.	N.D.
**IP-10/CXCL10**	(1.28±0.19) E+06	(5.27±1.83) E−04	0.41±0.08	(4.44±1.49) E+06	(4.22±1.38) E−03	0.95±0.08
**I-TAC/CXCL11**	(4.90±0.38) E+06	(7.49±0.04) E−02	15.3±1.1	(5.08±1.07) E+05	(5.73±1.44) E−02	118.3±53.3
**MCP-1/CCL2**	N.B.	N.B.	N.B.	N.B.	N.B.	N.B.
**MIP-1α/CCL3**	N.B.	N.B.	N.B.	N.B.	N.B.	N.B.
**MIP-1β/CCL4**	N.B.	N.B.	N.B.	N.B.	N.B.	N.B.
**RANTES/CCL5**	(1.36±1.19) E+03	(1.09±0.24) E−03	530±84	(5.31±1.88) E+03	(9.10±1.23) E−04	180±34

k_on_ association rate constant expressed in M^−1^ s^−1^; k_off_ dissociation rate constant expressed in s^−1^; K_D_ dissociation equilibrium (affinity) constant resulting from the ratio of k_off_ and k_on_, expressed in nM.

N.B. No binding signals were observed.

N.D. Kinetic parameters could not be determined.

Values represent means of two independent experiments ± standard deviations.

### COAM-anchored mouse GCP-2/CXCL6 potentiates fast *in* *vivo* neutrophil migration

**Figure 2:**
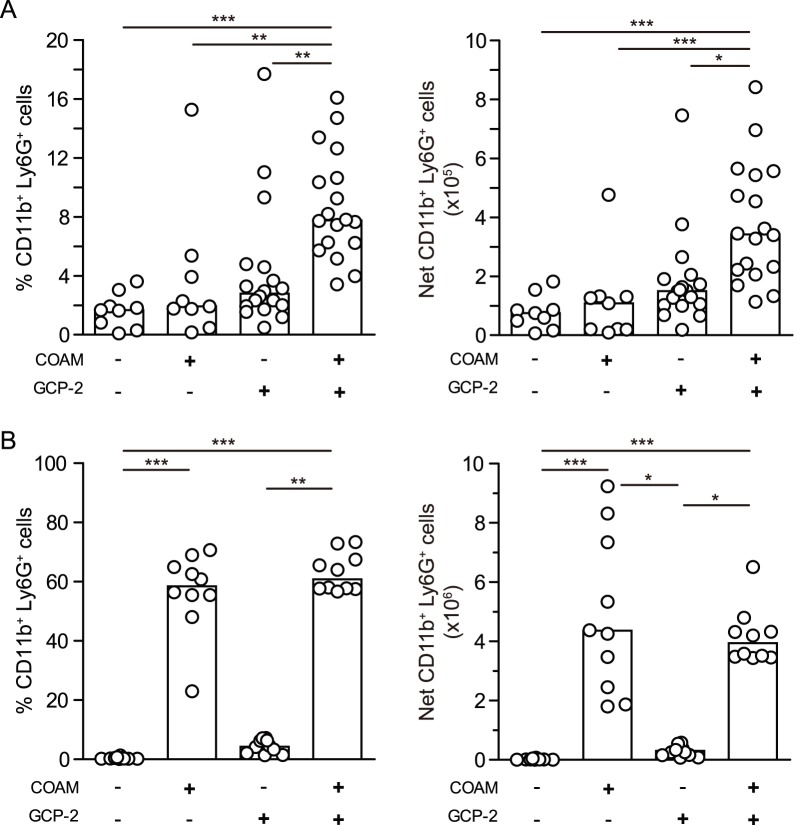
*In vivo* recruitment of neutrophils into the peritoneal cavity. Mice received an i.p. dose of 1-2_(7–98)_, or a mixture of both COAM (1 mg) and mouse GCP-2_(7–98)_ (100 ng). After 1 h (A) or 4 h post-treatment (B), peritoneal lavage fluids were collected and the percentages and absolute numbers of neutrophils, recognized as CD11b and Ly6G double positive cells, determined by FACS analysis, are shown. The net numbers of CD11b^+^ Ly6G^+^ cells were determined by multiplying the percentages of CD11b^+^ Ly6G^+^ cells with total peritoneal leukocyte counts. Histograms and dots represent group medians and spreading of individual data points from each mouse, respectively. *P<0.05, **P<0.01, ***P<0.001, as determined by Kruskal-Wallis test.

The interaction site of chemokines with their receptors is located at the chemokine aminoterminus, whereas chemokine binding to glycosaminoglycans is less well understood [11], [12]. If the binding of COAM would interfere with the chemokine receptor interaction, COAM should reduce chemotaxis. With the observation that COAM is a potent chemokine-binding molecule, we investigated whether this binding effect might potentiate the chemotaxis of leukocytes or rather inhibit this effect by blocking the chemokine aminoterminus. To this end, we studied the infiltration of neutrophils into the peritoneal cavity of mice following injection of mouse GCP-2_(9–78)_, alone or together with COAM. Intraperitoneal injection of COAM did not significantly change, within 1 h, the percentage and absolute numbers of recruited CD11b^+^ Ly6G^+^ cells, designated as being neutrophils (Fig. 2A). The percentage and absolute numbers of neutrophils slightly increased after 1 h upon injection of 100 ng GCP-2_(9–78)_. Moreover, a simultaneous injection of premixed COAM (1 mg) and GCP-2_(9–78)_ (100 ng) potentiated the chemotaxis of neutrophils toward the peritoneal cavity. Indeed, COAM-associated GCP-2_(9–78)_ significantly increased the percentages of infiltrated neutrophils as well as the net neutrophil numbers, when compared to the control group and to mice that received only GCP-2_(9–78)_. Furthermore, when analyzed after 4 h, a completely different picture emerged. Both the percentages as well as the net numbers of peritoneal neutrophils were significantly increased by COAM and no further potentiation of neutrophil chemotaxis was observed for GCP-2_(9–78)_ (Fig. 2B). This illustrated that the binding interactions of COAM with chemokines, observed *in* *vitro*, [16 and Fig. 1] can be translated to *in* *vivo* leukocyte migration, as the combination of COAM with GCP-2/CXCL6 potentiated the recruitment of neutrophils. These results suggested that COAM might mimic GAG functions, also *in* *vivo,* by interactions with (endogenous) chemokines that enhanced the migration of leukocytes at 4 h. Furthermore, this was in line with the idea that COAM binds to GCP-2/CXCL6 in a manner without interfering with the aminoterminal receptor-binding domain and signaling capacity of this chemokine. In this way, we could rule out that COAM inhibits local cell recruitment *in* *vivo* at the site of its injection.

### COAM enhances chemokine-induced neutrophil adhesion and transmigration *in* *vivo*

**Figure 3:**
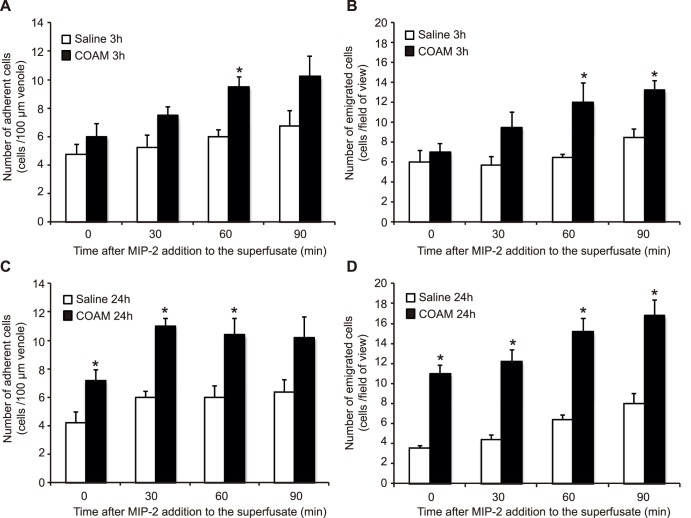
*In vivo* recruitment of neutrophils to the cremaster muscle. Mice received an intrascrotal dose of 0.2(A and B) or 24 (C and D) h prior to induction of anesthesia, surgical preparation of the cremaster muscle and onset of MIP-2/CXCL2 superperfusion. The number of adherent neutrophils (A and C) were quantified within a 100 µm length of venule, and the number of emigrated neutrophils within the field of view (B and D) were quantified prior to or following 30, 60 and 90 min of MIP-2/CXCL2 superperfusion. *P<0.05, **P<0.01, ***P<0.001, as determined by students’ t-test.

To reinforce the *in* *vivo* chemotaxis data, obtained with GCP-2_(9–78)_, we analyzed the effect of COAM injected intrascrotally prior to chemokine superperfusion of the cremaster muscle. In this case we used the neutrophil chemokine MIP-2/CXCL2 that has a lower affinity for COAM than GCP-2/CXCL6 (Table 1). At different time intervals (0, 30, 60, 90 min) after MIP-2/CXCL2 addition to the cremaster muscle superperfusate, neutrophil-endothelial cell interactions were registered through an intravital microscope, and the number of rolling neutrophils as well as their velocity (Tables 2 and 3), and the number of adherent (Fig. 3A, C) and emigrated (Fig. 3B, D) neutrophils were quantified. Pretreatment with COAM for 3 h did not significantly alter basal levels of neutrophil-endothelial cell interactions when compared to saline injections (Table 4). With time, following addition of MIP-2/CXCL2, the number of both adherent and emigrated neutrophils increased slightly in saline pretreated groups (Fig. 3 A–D). However, pretreatment with COAM intrascrotally 3 h prior to the experiment enhanced the chemoattracting ability of MIP-2/CXCL2 and significantly more neutrophils were adhering and emigrating compared to the saline treated group receiving MIP-2/CXCL2 (Fig. 3), while the numbers of rolling cells and their velocities were not changed (Tables 2 and 3). When COAM was administered 24 h prior to the experiments, the number of recruited cells were significantly increased already prior to addition of MIP-2/CXCL2, suggesting that COAM binds endogenous chemokines that retain the capacity to induce neutrophil recruitment from the cremasteric microcirculation (Fig. 3, Table 2). Following addition of MIP-2/CXCL2 to the superperfusate, the number of emigrated neutrophils was further increased, demonstrating that COAM potentiates transendothelial emigration to MIP-2/CXCL2. Even with a 20-fold lower affinity of MIP-2/CXCL2 for COAM, in comparison with GCP-2/CXCL6, COAM enhanced, rather than diminished, the local biological effect of MIP-2/CXCL2 on leukocyte recruitment in this second *in* *vivo* animal model.

**Table 2:** Effect of MIP-2/CXCL2 superperfusion of the cremaster muscle on number of rolling neutrophils (cells/min).

Time following MIP-2/CXCL2addition to the superperfusate	30 min	60 min	90 min
Saline 3 h	56±4	42±3	37±3
COAM 3 h	37±7	29±7	21±4
Saline 24 h	30±5	24±4	17±3
COAM 24 h	53±7*	43±5*	38±7*

*Numbers represent means ± SEM. *Indicates p<0.05 *versus* saline control.

**Table 3:** Effect of MIP-2/CXCL2 superperfusion on rolling neutrophil velocity (µm/s) in the cremaster muscle.

Time following MIP-2/CXCL2addition to the superperfusate	30 min	60 min	90 min
Saline 3 h	25±6	25±4	33±4
COAM 3 h	32±7	29±4	28±3
Saline 24 h	17±5	20±5	22±7
COAM 24 h	26±5	29±5	33±6

*Numbers represent means ± SEM.

**Table 4:** Numbers of neutrophil-endothelial cell interactions observed 30 min following surgical preparation of the cremaster muscle after 3 h pretreatment with saline or COAM.

	Saline treated mice	COAM treated mice
Number of rolling cells (cells/min)	64±8	41±5
Rolling cell velocity (µm/s)	19±3	21±5
Number of adherent cells (cells/min)	5±2	6±1
Number of emigrated cells(cells/field of view)	6±2	6±1

*Numbers represent means ± SEM.

### Intraperitoneal COAM affects neutrophil recruitment and NET formation in the inflamed liver

**Figure 4:**
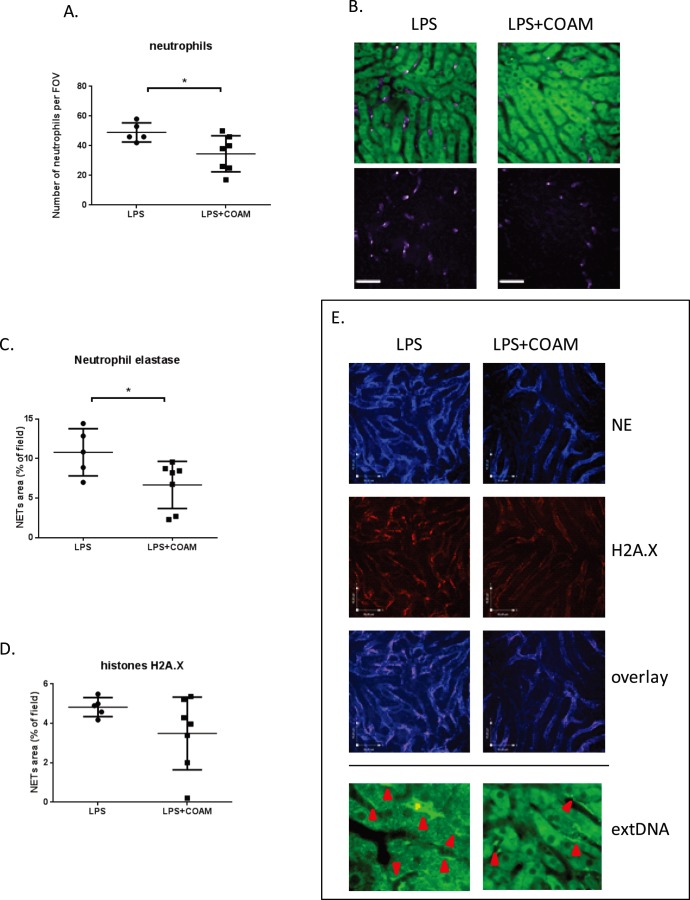
Effects of COAM co-treatment on LPS-induced systemic inflammation in the liver. (A) Co-application of COAM (2 mg/mouse) with intraperitoneally administrated LPS (1 mg/kg) decreases neutrophil infiltration to the liver at 4 h of inflammation; (B) representative images of neutrophils present in the liver sinusoids of LPS- and LPS plus COAM-treated mice (green cells – autofluorescent hepatocytes; 20x; scale bars represent 50 µm). Quantification of extracellular neutrophil elastase (C) and histone (D) within the livers of LPS and LPS+COAM-treated animals (mean area of staining per 20×FOV ± SD; scale bars represent 45 µm). Intravital visualization of NET deposition in the liver vasculature of LPS-treated and LPS plus COAM-treated mice (E). Staining for extracellular neutrophil elastase (NE) and histone illustrates clear deposition of these characteristic molecules of NETs in the liver after either treatment. In addition, overlay of histone and elastase staining is shown. Staining for extracellular DNA is presented with a higher magnification to clearly picture Sytox green deposition along the liver sinusoids; areas of the extDNA deposition are marked with red arrows. Neutrophil, elastase and histones were measured in five FOV/mouse, n = 5–7 animals per group; *P<0.05.

Both previously reported *in* *vivo* cell recruitment effects of COAM were studied locally, i.e. at the site of COAM injection. We next evaluated whether local COAM injection affected leukocyte counts in a distant organ. Intraperitoneal administration of LPS (endotoxinemia) leads to neutrophil recruitment to the liver microvasculature and the release of NETs that protect host cells from infection, as shown previously [25]. Here we visualized the liver microvasculature using spinning-disk confocal intravital microscopy (SD-IVM). In endotoxemic mice, 4 h after the administration of LPS, we observed 10-fold increased numbers of accumulated neutrophils (Fig. 4A) (per field 49,00±2,864 cells upon LPS treatment *versus* 4,00±3,61 cell in untreated mice; the latter group not shown). When COAM was injected in the peritoneal cavity together with LPS, the recruitment of neutrophils, which is known to be massive after ip injection of COAM [16], was significantly decreased in the liver, in comparison with the LPS-treatment group. This demonstrates that local injection of COAM may lead to a systemic effect in the liver (Fig. 4 A, B).

The injection of LPS also induced NET formation in the liver sinusoids (Fig. 4 C–E). We visualized NETs as structures composed of the extracellular DNA with attached histones and neutrophil elastase *in* *vivo*. To detect the presence of extracellular DNA (extDNA) within the liver vasculature, we intravenously infused the cell-impermeable DNA dye Sytox Green, and to undoubtedly confirm that these structures are NETs we intravenously applied fluorescently labeled antibodies specific for histone H2Ax (red) and neutrophil elastase (blue) and demonstrated that these NET-defining components colocalize (Fig. 4E). NETs, as revealed by both histone and elastase staining, were observed stably attached to the vessel walls of the liver (Fig. 4E). When we quantified the area covered by neutrophil elastase within the liver sinusoids, we detected a significant difference of 40% between mice treated with LPS only, and LPS co-administrated together with COAM. The presence of COAM led to significant decrease in the elastase staining (Fig. 4C). There was also a tendency to decreased histone levels (Fig. 4D). We also verified the presence of extDNA (Fig. 4E bottom) but this parameter was not quantified due to the fact that hepatocytes are autofluorecently green. Nevertheless, when comparing the images, there was a clear difference between the LPS- and LPS plus COAM-treated mice as in the latter ones less extDNA could be observed (Fig. 4E bottom). To confirm that Sytox Green specifically stained for extDNA and was useful to detect the NET-backbone, we intravenously infused DNase which completely dissolved the green staining along the liver sinusoids (not shown). In conclusion, these data demonstrated that intraperitoneal administration of COAM, which itself generates local recruitment of neutrophils [16], results in general effects to the extent that this infiltration supersedes the expected neutrophil migration to the liver as a distant organ.

## Discussion

We demonstrated here that COAM is an excellent probe to study local tissue-specific leukocyte recruitment and its systemic effects on leukocyte migration in distant organs. Neutrophil chemotaxis toward the site of COAM injection was originally associated with the presence of elevated amounts of the ELR^+^ CXC chemokine GCP-2/CXCL6 and binding to COAM, cell recruitment and virus destruction [16]. Nothing was known about effects of COAM on the expression and binding to chemokines, other than GCP-2/CXCL6. Furthermore, whereas the effects of COAM on local injection are evident [16], [18], COAM may also exert effects on distant organs [17]. To study distant effects, we used here an animal model of LPS-induced recruitment of neutrophils to the liver and local neutrophil extracellular traps (NET) formation, and imaged in real time immunological events occurring upon COAM treatment with confocal intravital microscopy. Whereas intraperitoneal injection of COAM had the expected local effects on neutrophil recruitment [16], it also reduced significantly the LPS-induced influx of neutrophils to the liver as well as the subsequent formation of NETs by these cells. This illustrates that local COAM injection is so potent that it generates systemic effects such as decreasing the numbers of leukocytes in the liver or the central nervous system [17]. In these distant organs, COAM thus might possess anti-inflammatory properties. Our studies with three different animal models thus indicate that COAM is an excellent probe to study also leukocyte recruitment by endogenous chemokines *in* *vivo*.

Another aspect was to evaluate whether other, if not all, chemokines are affected by COAM as a way to broaden our understanding of polycarboxylates on leukocyte recruitment *in* *vivo*. It is clear that COAM induced chemokines in cell-specific ways and bound chemokines with varying affinities. It has previously been shown that the peritoneal mesothelium and resident macrophages, but also peritoneal fibroblasts, represent key sources of chemokines, such as KC/CXCL1, MIP-2/CXCL2, MCP-1/CCL2, RANTES/CCL5 and IP-10/CXCL10, upon stimulation with cytokines, such as TNF-α, IL-1β and IFN-γ. These cells thus may provide endogenous chemokines in the intraperitoneal environment [5], [7], [24], [26], [27]. Furthermore, neutrophils and other leukocytes also produce an array of chemokines, including neutrophil chemotactic ELR+CXC chemokines and T cell attracting chemokines MIG/CXL9, IP-10/CXCL10 and I-TAC/CXCL11 [5], [28], whereas endogenous MIP-2/CXCL2 may originate from resident macrophages or other peritoneal cells [29].

We showed that COAM is a potent chemokine-binding molecule and this fact may explain the observed *in* *vivo* cell recruitment results. Consistently, we observed binding of COAM to the neutrophil chemoattractants GCP-2/CXCL6, KC/CXCL1 and MIP-2/CXCL2. The affinities for GCP-2/CXCL6, KC/CXCL1 and MIP-2/CXCL2 were in the nM-range and similar to binding affinities determined for heparan sulphate. Similarly, IP-10/CXCL10 was found to be a chemokine with high affinity for COAM as well as for heparan sulphate, whereas the tested CC chemokines RANTES/CCL5 and MIP-1α/CCL3, MIP-1β/CCL4 or MCP-1/CCL2 displayed only weak or no binding. Due to the negatively charged nature of GAGs and the highly basic character of most chemokines (isoelectric points between pI 9–10, except for MIP-1α/CCL3 or MIP-1β/CCL4, see Table 5), interactions between chemokines and GAGs were understood to depend on non-selective electrostatic forces. However, the discovery of distinct GAG-binding epitopes added a degree of specificity to the level of regulation of chemokine action [11]. Mutations in GAG-binding sites of MCP-1/CCL2, MIP-1β/CCL4 and RANTES/CCL5 disturb chemotactic activity *in* *vivo.* However, chemotaxis *in* *vitro* is not affected. Moreover, the formation of higher-order chemokines and their oligomerization on GAGs is also pivotal for their *in* *vivo* function, further underlining the importance and absolute requirement of GAG binding to chemokines *in* *vivo* [30]. The existence of differential kinetics of chemokine-GAG interactions may be an important mechanism by which distinct chemokine gradients are orchestrated *in* *vivo*. For instance, following KC/CXCL1 and MIP-2/CXCL2 instillation in lungs, quantitative and temporal differences in pulmonary neutrophil recruitment are based on differences in association and dissociation rates of chemokines with heparan sulphate [31]. Complementary to the suggested endogenous chemokine-binding and –presenting functions of COAM, the in vivo mechanism of action of COAM may also involve the presentation of (inactive) matrix-bound chemokines or the prolongation of the chemokine actions/half-lives.

**Table 5:** Theoretical isoelectric points and carboxyterminal amino acid sequences of mouse chemokines.

Mouse chemokine	Isoelectric point (theoretical)	COOH-terminal amino acid sequence	Pubmed entry number
MIG (CXCL9)	10.62	- KQKRGKKHQKNMKNRKPKTPQSRRRSRKTT	P18340
I-TAC (CXCL11)	10.11	- RQRCLDPRSKQARLIMQAIEKKNFLRRQNM	Q9JHH5
IP-10 (CXCL10)	10.05	- NDEQRCLNPESKTIKNLMKAFSQKRSKRAP	P17515
MCP-1 (CCL2)	9.81	- LTRKSEANASTTFSTTTSSTSVGVTSVTVN	P10148
MIP-2 (CXCL2)	9.30	- LKGGQKVCLDPEAPLVQKIIQKILNKGKAN	P10889
GCP-2_9–78_ (CXCL6)	9.21	- KNQKEVCLDPEAPVIKKIIQKILGSDKKKA	P50228
KC (CXCL1)	9.10	- TLKNGREACLDPEAPLVQKIVQKMLKGVPK	P12850
RANTES (CCL5)	8.76	- VVFVTRRNRQVCANPEKKWVQEYINYLEMS	P30882
MIP-1β (CCL4)	5.64	- VVFLTKRGRQICANPSEPWVTEYMSDLELN	P14097
MIP-1α (CCL3)	5.14	- IFLTKRNRQICADSKETWVQEYITDLELNA	P10855

Basic amino acids (arginine, R; lysine, K) are underlined.

Aside electrostatic forces, the presence of GAG-binding epitopes on chemokines provides a certain degree of selectivity [11]. All tested chemokines with theoretical pI values between 8.5 and 11 and containing 5 to 15 basic amino acids in their carboxyterminus were found to bind COAM as well as heparan sulphate. The low abundance of positively-charged amino acids in its carboxyterminus together with a high C-terminal serine/threonine content might explain why mouse MCP-1/CCL2 did not bind to COAM or heparan sulphate. For clarity, human MCP-1/CXCL2 has different biochemical characteristics than mouse MCP-1/CXCL2, mainly by considerable differences at its carboxyterminus, the supposed interaction site with COAM. For instance and in comparison with the other studied mouse chemokines, mouse MCP-1/CXCL2 contains only two basic amino acids in its carboxyterminus (Table 5). This constitutes a plausible explanation for differences in heparin sulphate (and COAM) binding of human MCP-1/CXCL2 [32], [33] *versus* mouse MCP-1/CXCL2 (this study). Likewise, the low theoretical pIs of MIP-1α/CCL3 and –β/CCL4, respectively 5.14 and 5.64, together with few basic carboxy-terminal amino acids likely explain our negative chemokine binding results for COAM and heparan sulphate. In view of the chemical structure of COAM [19], chemokine binding to COAM might protect these chemokines from proteolytic degradation. Protection from proteolysis has been demonstrated in the case of interaction between heparin and eotaxin [12] and between heparan sulphate and IL-8 [34] or SDF-1 [35].

Soluble GAGs, in contrast to cell surface- or extracellular matrix-associated GAGs, when forming complexes with chemokines, inhibit chemokine receptor activation by competition for chemokine binding, resulting in inhibition of leukocyte responses [36], [37]. In sharp contrast to these findings with heparin, our results suggest that COAM, as a soluble molecule displaying GAG mimetic properties, does not inhibit but instead stimulates chemokine function by binding to chemokines, and in particular potentiates neutrophil chemotaxis toward GCP-2/CXCL6 and MIP-2/CXCL2 *in* *vivo*. COAM, by its high affinity for (neutrophil) chemokines might act like a sponge and bind local and systemic chemokines in such a way that they retain their chemotactic activity. In this way, the majority of leukocytes is recruited to the COAM injection site and thus might displace leukocytes from distant organs, as was here observed in the liver tissue. Although alternative explanations are possible, we suggest that COAM, by its repetitive structure, might bind and present endogenous chemokines side-by-side in a multivalent way and with the chemokine receptor-binding face exposed, in order to support efficient cell recruitment. This recruitment phenomenon by COAM is so potent that it also has systemic effects, as previously shown on the central nervous system [17] and, in this study, on the liver.

In conclusion, we have shown that the polysaccharide derivative COAM formed a binding complex with chemokines, which in turn influenced chemokine localization and selectivity of leukocyte responses. As evidenced here in three *in* *vivo* models, binding of chemokines to COAM affected neutrophil migration *in* *vivo*. The insights obtained by this study about the relative binding selectivity of COAM for specific chemokines may be exploited to redirect the migration of specific leukocytes *in* *vivo*. In this way, COAM is an interesting molecular probe for chemokine-mediated immunomodulation and stands as a first example of an effective GAG mimetic, retaining chemotactic functionality of bound chemokines.
